# Portable electrochemical immunosensor for point-of-care testing of ovarian cancer biomarker HE4 in commercial human blood serum samples

**DOI:** 10.1007/s00216-026-06457-7

**Published:** 2026-03-29

**Authors:** Merve Yılmaz Çılçar, Melike Bilgi Kamaç

**Affiliations:** Chemistry Department, Faculty of Science, Çankırı Karatekin University, Çankırı, 18100 Türkiye

**Keywords:** Portable HE4 immunosensor, Ti_3_C_2_, Ti_3_C_2_-COOH, AuNP, Ovarian cancer, Point-of-care testing

## Abstract

Ovarian cancer ranks as the seventh most prevalent malignancy among women globally. Human epididymis protein 4 (HE4) is an FDA-approved tumor biomarker for the early detection of malignant ovarian cancer. In this work, a portable, electrochemical HE4 immunosensor was fabricated for the rapid, sensitive, and practical analysis of HE4 levels in commercial human blood serum. In the preparation of immunosensors, nanomaterials such as Ti_3_C_2_, Ti_3_C_2_-COOH, and gold nanoparticles (AuNPs), as well as disposable screen-printed carbon electrodes, were used. The influence of nanomaterials on the electrochemical efficiency of electrodes and immunosensors was examined. The materials of Ti_3_C_2_, Ti_3_C_2_-COOH, and AuNP, and the modified electrodes with these materials, were characterized by FE-SEM-EDX, FTIR, and XRD. Optimization studies and evaluation of analytical performance of the HE4 immunosensor were conducted using a portable electrochemical reader with DPV. The linear range and the detection limit of HE4 immunosensors were determined to be 5–250 pM and 0.34 pM, respectively. Stability was observed for 60 days, and shelf life was assessed for 24 weeks. The selectivity of HE4 immunosensors was tested in the presence of other ovarian cancer biomarkers and found to be highly selective for HE4. In repeatability tests, HE4 immunosensors responded to seven repetitions (RSD: 3.38%, *n* = 7). Method validation of the immunosensor platform was confirmed by ELISA. The HE4 levels in commercial human blood serum were directly measured on-screen in picomolar (pM) (immunosensor setup time: 105 min; antibody-antigen-BSA incubations) and a portable electrochemical reader (device reading time: 20–30 s). The developed HE4 immunosensors are potential candidates in point-of-care testing.

**Graphical abstract:**

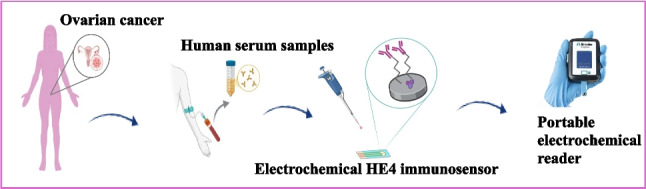

**Supplementary Information:**

The online version contains supplementary material available at 10.1007/s00216-026-06457-7.

##  Introduction

Global 2022 data show that among female cancer types, ovarian cancer ranks 7th worldwide by prevalence (6.7%) and 6th by mortality (4%) [1]. The high mortality rate is attributable to the late-stage diagnosis of ovarian cancer. Ovarian cancer, called the “silent killer,” does not show any noticeable symptoms until the late stages, and there is no reliable screening method yet [2–5]. Although most women with early-stage ovarian cancer can be cured with appropriate treatments, most women with late-stage ovarian cancer experience multiple recurrent episodes with progressively shorter disease-free intervals. Manual pelvic examinations, transvaginal sonography (TVS), and cancer antigen 125 (CA125) levels are commonly used for the early diagnosis of ovarian cancer. However, a manual pelvic examination is inadequate for distinguishing between benign and malignant lesions [6]. TVS provides increased accuracy in detecting abnormalities in ovarian volume and morphology. However, it has low sensitivity for distinguishing benign from malignant ovarian tumors [7]. Therefore, early diagnosis of ovarian cancer is a crucial research area [8]. Detection of low levels of biomarkers in human serum is crucial for the diagnosis and monitoring of cancer in the early stages [2, 9]. Before 2008, CA125 was the only biomarker authorized by the Food and Drug Administration (FDA) for ovarian cancer. Although CA125 is elevated in 80% of women with advanced ovarian cancer, it is elevated in only 50% of women with stages I and II ovarian cancer [10]. Additionally, CA125 levels are elevated in some benign gynecological diseases and in malignant non-gynecological diseases [11]. Therefore, other biomarkers are needed for early diagnosis and monitoring of ovarian cancer. Among various biomarkers, human epididymis protein 4 (HE4) stands out as the most intriguing and is an FDA-approved biomarker. While HE4 is elevated in all stages of ovarian cancer, it is a more sensitive biomarker than CA125 in stage I, which is important for early diagnosis [2, 12]. Several analytical methods are available for detecting biomarkers, including enzyme-linked immunosorbent assay (ELISA), radioimmunoassay, immunofluorescence, and chemiluminescence. These conventional methods have drawbacks, including poor repeatability, time inefficiency, high cost, and the need for skilled staff. Unlike older approaches, electrochemical biosensors have garnered significant interest owing to their rapid response, high sensitivity and selectivity, cost-effectiveness, and potential for downsizing [13–15]. Antibody-based electrochemical biosensors offer superior performance compared to conventional analytical techniques in detecting cancer biomarkers at low concentrations with high selectivity [14–17].

Modifications of electrodes with nanomaterials are crucial for the formation of biosensing interfaces [18, 19]. Ti_3_C_2_ (MXene), a 2D material similar to graphene, has gained increasing popularity in electrochemical sensor systems recently due to its high surface area and functional groups [20]. Research indicates that incorporating Ti_3_C_2_ enhances the electrode’s surface area and improves its electrochemical performance. For example, researchers utilized Ti_3_C_2_-modified screen-printed electrodes (SPEs) to simultaneously analyze acetaminophen and isoniazid. Ti_3_C_2_ was found to exhibit electrocatalytic activity, high surface area, and electronic conductivity [21]. Lei et al. prepared a wearable enzyme-based biosensor and determined glucose and lactate using Ti_3_C_2_/Prussian blue (Ti_3_C_2_Tx/PB). It was emphasized that the presence of Ti_3_C_2_Tx/PB extended the shelf life of enzyme-based biosensors [22]. In studies conducted after 2020, it was seen that the functionality of Ti_3_C_2_ was used. In one of these studies, an electrochemical Amyloid beta 42 biosensor was designed based on a composite of delaminated Ti_3_C_2_ (d-Ti_3_C_2_Tx) and multi-walled carbon nanotubes (MWCNTs) containing molecularly imprinted polymers on a glassy carbon electrode (GCE). It was reported that the MWCNT/d-Ti_3_C_2_Tx increased the electrical conductivity of the biosensor [23]. Au@Ti_3_C_2_@PEI-Ru(dcbpy)_3_^2+^ nanocomposite was used in the transducer part of the electrochemiluminescence biosensor prepared for the analysis of severe acute respiratory syndrome coronavirus 2, and it was emphasized that Ti_3_C_2_ increased the surface area of the biosensor and exhibited good stability [24]. In another study, indium tin oxide (ITO) was modified with Ti_3_C_2_ and TiO_2_, respectively, and utilized in the preparation of an electrochemical immunosensor for detecting the epithelial cell adhesion molecule (EpCAM). It was reported that the EpCAM immunosensor enhanced the electron transfer rate and possessed a large surface area due to the incorporation of Ti_3_C_2_ and TiO_2_ [25]. In a study in which Ti_3_C_2_ nanolayers were synthesized and used as electrodes in humidity sensors, the authors reported that the cost of the sensor was reduced [26]. Biomarkers in serum were analyzed using screen-printed carbon electrodes (SPCEs) modified with MXene and gold nanoparticles (AuNPs). MXene and AuNP were reported to enhance the performance of the biosensor [27]. From these results, it was understood that the performance of biosensors produced by Ti_3_C_2_ and functionalized Ti_3_C_2_ could be increased. In this work, we compared the electrochemical efficiency of SPCE by modifying them with Ti_3_C_2_ and carboxyl-functionalized Ti_3_C_2_ (Ti_3_C_2_-COOH). We also modified SPCE/Ti_3_C_2_ and SPCE/Ti_3_C_2_-COOH electrodes with AuNPs to investigate their interactions with Ti_3_C_2_ and Ti_3_C_2_-COOH, and their use in HE4 immunosensors for the first time.

In recent years, various electrochemical and optical biosensing methodologies have been presented for the determination of HE4. Most immunosensors are designed in a sandwich format, which requires two different antibodies to bind specifically to the antigen. All these studies utilize bioassay platforms that comprise magnetic micro- or nanoparticles and nanomaterials for signal amplification or biomolecular enrichment [28]. Despite their sensitivity, the labeled HE4 immunosensors reported in the literature have drawbacks, including being time-consuming and costly [29–34]. Label-free electrochemical immunosensors are practical and relatively low-cost [2]. They also allow for more sensitive and versatile detection [35]. Few studies have investigated label-free electrochemical HE4 biosensors. For instance, Yuan et al. fabricated a label-free HE4 immunosensor based on localized surface plasmon resonance using AgNPs and anti-HE4 and successfully determined HE4 in the serum of ovarian cancer patients. However, this method is time-consuming and costly [36]. Tan et al. developed a photoelectrochemical immunosensing platform based on exfoliated tungsten disulfide nanolayers functionalized with an ionic liquid (IL-WS2) and hollow gold nanospheres, on which anti-HE4 was immobilized. However, the IL-WS2 used in the study is laborious because it requires complex pretreatment [37]. In another study, researchers designed a label-free biosensing platform comprising fullerene C60, a nanoporous anodic alumina membrane, and a GCE to determine HE4 levels. Since GCEs are not suitable for point-of-care testing (POCT), the developed HE4 immunosensor is impractical [38]. Szymanska et al. designed a surface plasmon resonance-based label-free HE4 immunosensor on gold chips and successfully determined HE4 concentrations in the linear range of 2–120 pM, with a detection limit of 2 pM. The high price of the gold chip used in this study, however, highlights some of its drawbacks [39]. In a study in which heterostructured AuNPs/CdS NS were formed on the surface of a fluorine-doped tin oxide electrode by two-stage electrodeposition and used to prepare photoelectrochemical (PEC) and electrochemical (EC) dual-mode HE4 immunosensors, HE4 analysis exhibited good linear ranges in PEC and EC modes. The dual-mode method is laborious [40]. Vural et al. prepared a label-free HE4 immunosensor by forming -OH groups on the surface of ITO electrodes and immobilizing anti-HE4 after treatment with 3-aminopropyltriethoxysilane and glutaraldehyde, and they then analyzed HE4 in blood serum using impedimetric analysis. The developed HE4 immunosensor has a wide linear range but is not suitable for POCT because it employs a triple-electrode system [41].

POCT enables users to perform analyses quickly, economically, and without specialized expertise, regardless of location or time [2, 42]. Integrating POCT devices with biosensors facilitates analysis that is straightforward, rapid, and comparatively economical, particularly in healthcare, food quality assessment, and environmental surveillance [2, 43, 44]. Practical, economical, and disposable electrodes, such as SPEs, are highly suitable for POCT devices [45–48]. The label-free HE4 immunosensors were prepared in our previous studies and are suitable for POCT. In our 2023 study, Anti-HE4 was immobilized onto a self-assembled monolayer formed from 3-mercaptopropionic acid on the surface of SPCEs modified with reduced graphene oxide, polythionine, and AuNPs, and HE4 was analyzed using three electrochemical methods (CV, DPV, and EIS). HE4 levels in commercial human blood serum were measured by DPV using a portable electrochemical reader [2]. In 2023, our working group conducted a simultaneous analysis of CA125 and HE4 with immunosensors fabricated using dual SPCEs and employing DPV and SWV. In both studies, the ROMA score used to assess ovarian cancer risk in both studies was calculated from CA125 and HE4 analysis results [2, 49].

In this study, we extended our previous work by examining not only HE4 analysis in commercial human blood serum but also the optimal working parameters of HE4 immunosensors. All stages of the analytical characterizations were performed using a portable electrochemical reader. This study not only demonstrates the potential of SPEs for use in POCT but also guides researchers by showing that fast and practical biosensors can be developed using portable electrochemical readers and SPEs. In our study, rapid and practical HE4 immunosensors were fabricated using SPCE/Ti_3_C_2_/AuNP and SPCE/Ti_3_C_2_-COOH/AuNP. The synergistic combination of Ti_3_C_2_-COOH and AuNPs significantly enhanced the electrical conductivity of the electrode, providing a large electroactive surface area. This combination demonstrated superior performance in key evaluation parameters of immunosensors, including stability, shelf life, and reproducibility. For the first time, HE4 levels in commercial human blood serum were read directly from the screen in pM concentrations in just 20–30 s (device reading time) using HE4 immunosensors and portable electrochemical readers.

## Experimental

### Chemicals

Chloroauric acid (HAuCl_4_), bovine serum albumin (BSA), potassium hexacyanoferrate (K_3_Fe(CN)_6_), potassium hexacyanoferrite (K_4_Fe(CN)_6_), N-(3-dimethylamino propyl)-N′-ethyl carbodiimide (EDC), N-hydroxysuccinimide (NHS), human serum (from male AB clotted whole blood, H6914), 6-mercapto hexanol (6-MHA), were obtained from Sigma-Aldrich (USA). Monoclonal antibody HE4/WFDC2 and recombinant human HE4/WFDC2 protein were supplied by Novus Biologicals, USA (Ti_3_AlC_2_) (purity 98%) was obtained from Aaron Chemicals. For the method comparison, ELISA kits with code FY-EH4147 for HE4 analysis in commercial blood serum were obtained from Eıyve. Chloroacetic acid (ClCH_2_COOH), ethanol (EtOH), dimethyl formamide (DMF), potassium chloride (KCl), potassium dihydrogen phosphate (KH_2_PO_4_), potassium hydrogen phosphate (K_2_HPO_4_), and hydrochloric acid (HCl) were obtained from Merck. Hydrofluoric acid (HF, 40%) was supplied by Isolab chemicals. To make a phosphate buffer solution (PBS), K_2_HPO_4_, KH_2_PO_4_, and KCl were utilized. All of the solutions used were prepared with ultrapure water (Millipore, 18 MΩ cm).

### Instruments

The voltammetric analysis was performed using a Bipotentiostat/Galvanostat μStat 400 obtained from Methrom DropSens (Oviedo, Spain) and operated by a PC running DropView 800 software. Electrochemical impedance spectroscopy (EIS) measurements were performed using a Gamry Potentiostat/Galvanostat, Reference 1010E (Gamry Instruments, Warminster, USA), with data acquired on a computer running EChem Analyst. The DropStat Electrochemical Reader, a handheld device, and the disposable SPCEs were purchased from Methrom DropSens (Oviedo, Spain, ref. DRPX1110). SPCEs consist of a three-electrode system with working (WE) and counter electrodes of carbon and a reference electrode of silver. For chemical and morphological characterization, a Perkin Elmer Fourier-transform infrared (FT-IR), a ZEISS Gemini 1 Field Emission Scanning Electron Microscope (FE-SEM), and a PANalytical Empyrean X-ray Diffraction (XRD) device were used, respectively. ELISA absorbance values were measured using a microplate reader (BioTek ELx800) at 450 nm.

### Electrochemical measurements

Electrochemical characterizations were carried out with cyclic voltammetry (CV), differential pulse voltammetry (DPV), and electrochemical impedance spectroscopy (EIS) in redox probe solution (5 mM redox probe solution: K_3_Fe(CN)_6_/K_4_Fe(CN)_6_ in 1 M KCl). DPV measurements were performed using a portable handheld electrochemical reader in a 1 mM redox probe solution. Metrohm Dropsens software for applying the DPV method was installed on the Dropstat, and the peak current height (in µA) was read from the Dropstat screen. EIS measurements were carried out over a frequency range of 50.000 Hz to 0.05 Hz. The following parameters were used for CV measurements: potential ranges of −0.5 V to + 0.8 V at 50 mV s^−1^. DPV measurement parameters were pulse potential 70 mV, pulse time 0.1 s, step potential 5 mV, and scan rate 5 mV s^−1^. Optimization of the operating parameters (antibody concentration, incubation times of antibody and antigen) and determination of analytical parameters (linear range, repeatability, reproducibility, stability, etc.) for HE4 immunosensors (sections) were performed using the DPV technique on a DropStat handheld electrochemical reader (Metrohm Dropsens). To perform DPV measurements with the device, software was installed on the DropStat by Metrohm Dropsens, and the DPV analysis results were displayed on the screen in µA. Since DPV measurements with DropStat could not exceed 200 µA, measurements were performed in a 1 mM redox probe solution. The measurement parameters of the DPV technique run on DropStat are: Starting potential: −0.5 V, Ending potential: + 0.4 V, Pulse potential: 0.07 V, Pulse time: 100 ms, Scan rate: 0.01 V/s, Step potential: 0.005 V. The procedure used to measure HE4 levels in commercial blood serum samples using DropStat: obtained from experiments in this study were sent to Metrohm Dropsens. The company prepared USB cards for calibration, installed the software, and sent them to us for use in actual sample analyses. HE4 levels in commercial blood serum were measured by directly reading HE4 concentrations in pM from the DropStat screen using the DPV method.

### Preparation of Ti_3_C_2_ and Ti_3_C_2_-COOH

**Scheme 1:**
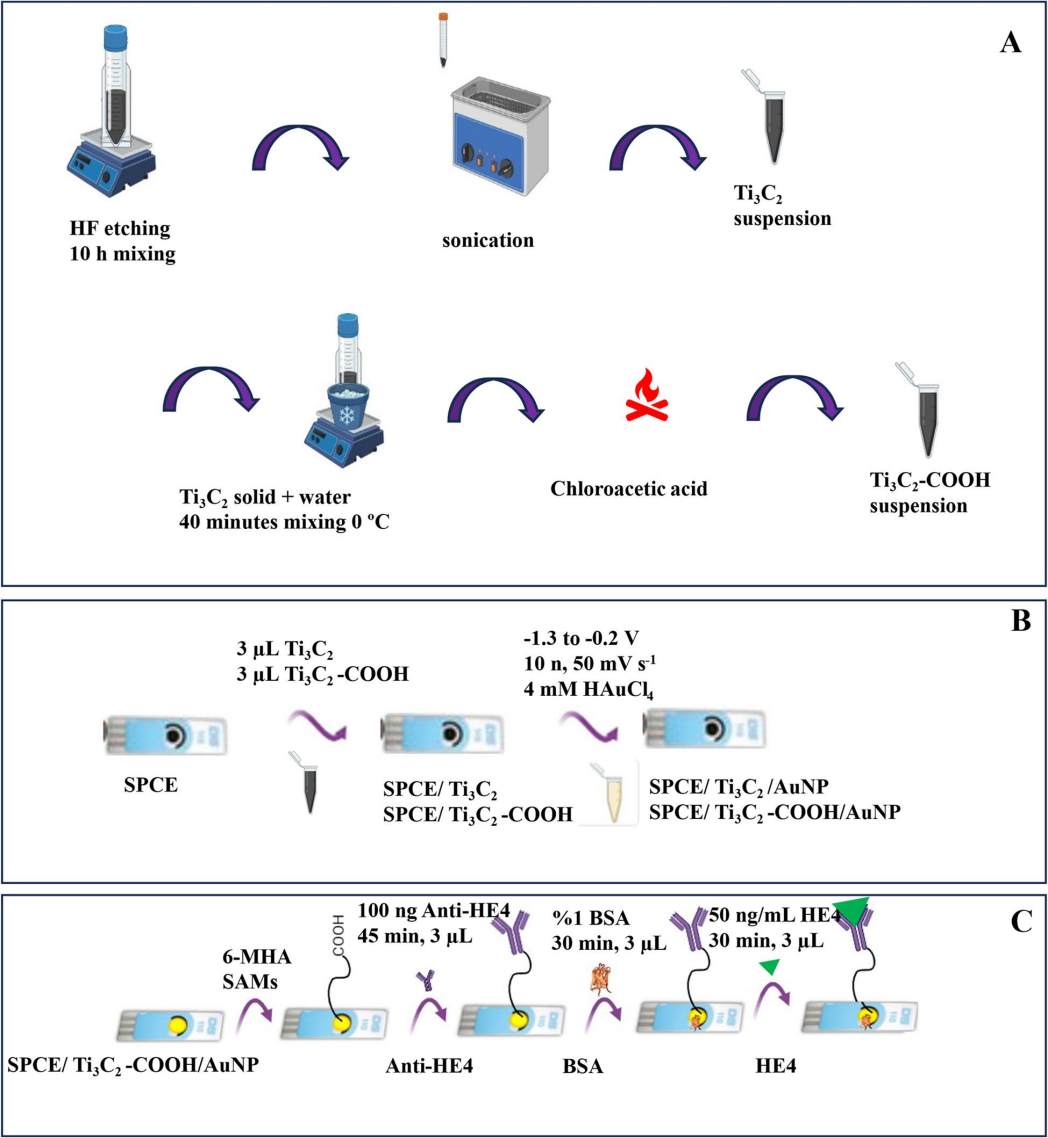
The preparation of the Ti_3_C_2_ and Ti_3_C_2_-COOH (**A**), the construction stage of the modified electrodes (**B**), and the HE4 immunosensor (**C**)

To prepare Ti_3_C_2_T_x_, 1 g of Ti_3_AlC_2_ was weighed and stirred in 40% HF (50 mL) for 10 h. The resulting suspension was washed several times with ultrapure water, centrifuged (4000 rpm for 30 min), and the separated solid was dried (under a vacuum at 50 °C) [50, 51]. To generate -COOH groups at the Ti_3_C_2_ ends, firstly, 300 mg of Ti_3_C_2_ powder was combined with 300 mL of deionized water at 0 °C and subjected to mechanical agitation for 40 min. Then, 5 g of chloroacetic acid was added slowly to the mixture. A 32 mL solution of NaOH (6.25 mol/L) was introduced to eliminate unreacted groups. The reaction was left at 60 °C for 3 h to complete. The resulting product was washed several times to remove impurities. Finally, it was freeze-dried at a low temperature (−50 °C) to obtain Ti_3_C_2_-COOH [52]. To prepare Ti_3_C_2_ and Ti_3_C_2_-COOH suspensions, 1 mg of solid was weighed, mixed with 1 mL of a 1:1 DMF-H_2_O solution, and sonicated for 3 h. Preparation steps of Ti_3_C_2_ and Ti_3_C_2_-COOH are given in Scheme 1A.

### Preparation of the modified electrode

3 µL of the Ti_3_C_2_ and Ti_3_C_2_-COOH suspensions was dropped onto the working electrode (WE) surfaces of the SPCEs and left until dry (Maleski vd., 2017; Gao vd., 2020; Mohammadniaei vd., 2020). To form AuNP on SPCE/Ti_3_C_2_ and SPCE/Ti_3_C_2_-COOH, HAuCl_4_ solution (50 µL of 6 mM in pH 7.0 PBS) was dropped onto WE, and 15 cycles of cyclic voltammetry (CV) (−1.3 V to −0.2 V, 50 mV s^−1^) were carried out [2, 49, 53–55]. The preparation steps for the modified electrodes are illustrated in Scheme 1B.

### Fabrication of the HE4 immunosensor

First, to form self-assembly monolayers (SAMs) using SPCE/Ti_3_C_2_/AuNP and SPCE/Ti_3_C_2_-COOH/AuNP, 3 µL of 6-mercaptohexanoic acid (6-MHA) (100 mM in ethanol) was dropped onto the WE surface and left for 18 h [2, 49, 56]. The WE was then treated with 3 µL of the EDC/NHS solution (0.6 mM/0.1 mM) and incubated for an hour [2, 49, 55]. After activation with 6-MHA and EDC/NHS, the WE was treated with 100 ng (3 µL) of anti-HE4 solution for 45 min. Then, 3 µL of BSA (1.0% (w/v)) was applied to the WE and left to stand for 30 min. Subsequently, 3 µL of BSA (1.0% (w/v)) was applied to the WE and incubated for 30 min. Finally, 3 µL of the HE4 solution was deposited onto the WE and incubated for 30 min. The fabrication steps for HE4 immunosensors are shown in Scheme 1C.

## Results and discussion

### Morphological and chemical characterizations

#### SEM-EDX

**Fig. 1:**
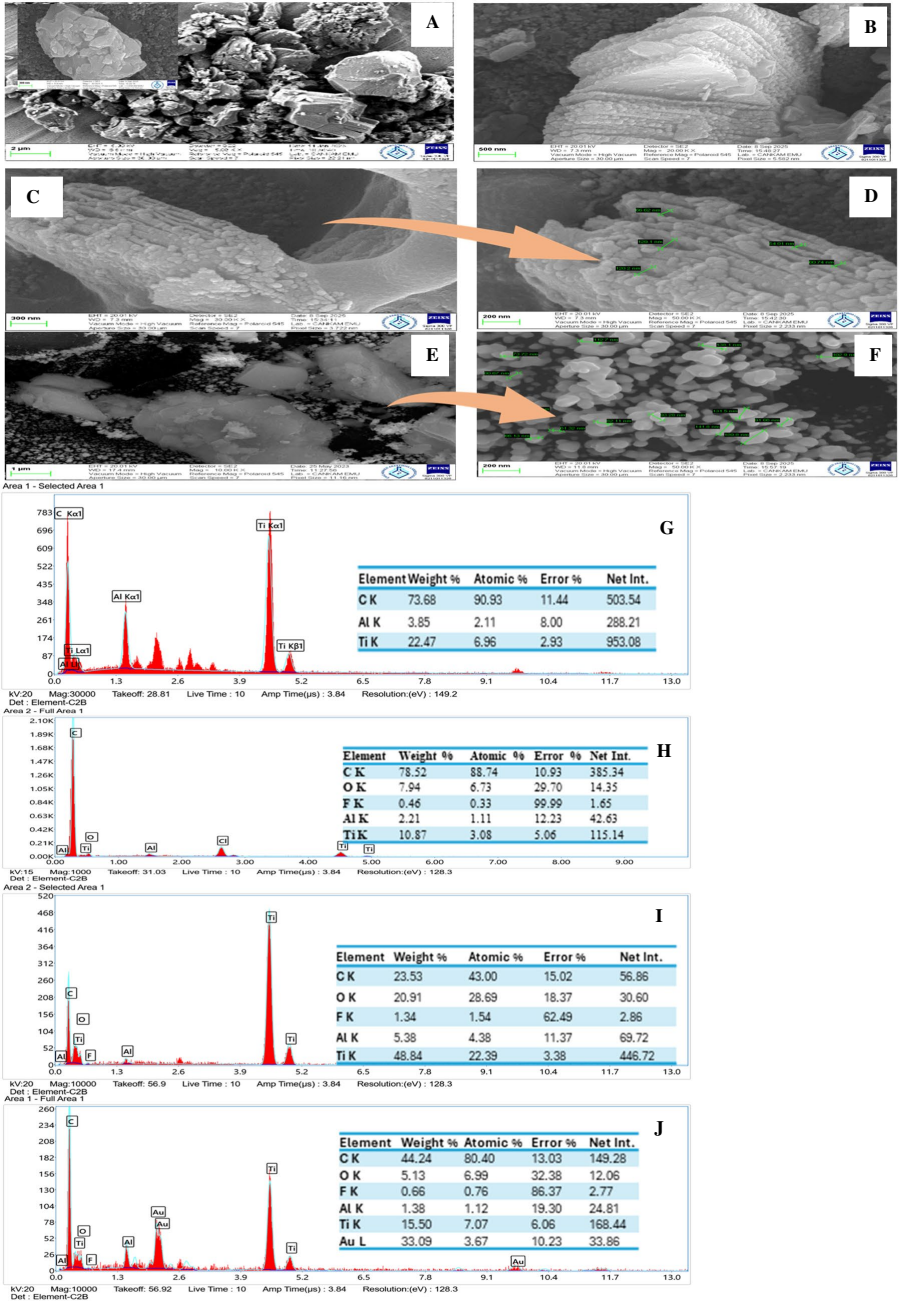
SEM images of Ti_3_AlC_2_ (**A**), Ti_3_C_2_ (**B**), SPCE/Ti_3_C_2_-COOH (**C**, **D**), and SPCE/Ti_3_C_2_-COOH/AuNP (**E**, **F**). EDX spectra of Ti_3_AlC_2_ (**G**), Ti_3_C_2_ (**H**), SPCE/Ti_3_C_2_-COOH (**I**), and SPCE/Ti_3_C_2_-COOH/AuNP (**J**)

SEM images were taken for the morphological characterization of the Ti_3_AlC_2_, Ti_3_C_2_, SPCE/Ti_3_C_2_-COOH, and SPCE/Ti_3_C_2_-COOH/AuNP, and are given in Fig. 1A–J. The SEM image of the Ti_3_AlC_2_ in Fig. 1A shows macroparticles in the form of agglomerates, while Fig. 1B shows Ti_3_C_2_s in the form of macro- and mesoparticles. In the SEM images of SPCE/Ti_3_C_2_-COOH (Fig. 1C) and zoomed-in SPCE/Ti_3_C_2_-COOH electrodes (Fig. 1D), the existence of multilayers of Ti_3_C_2_-COOH is evidenced [57]. The spherical shape of the AuNPs was observed in some places in the SEM image of the SPCE/Ti_3_C_2_-COOH/AuNP (Fig. 1E, 1) [53–55]. The particle sizes of Ti_3_C_2_-COOH and AuNP were measured using SEM images and determined to be in the range of 55–120 nm and 60–200 nm, respectively. The presence of Ti and Al is observed in the EDX spectrum of the Ti_3_AlC_2_ in Fig. 1G, while the presence of Ti and a tiny amount of Al is observed in Ti_3_C_2_ (Fig. 1H). In the EDX spectra of the SPCE/Ti_3_C_2_-COOH (F1g. 1I) and SPCE/Ti_3_C_2_-COOH/AuNP (Fig. 1J), the presence of C and Ti and C, Ti, and Au [54, 55, 57] in the structures is observed, respectively. According to SEM-EDX results, SPCE/Ti_3_C_2_-COOH/AuNP electrodes were successfully prepared.

#### FTIR

**Fig. 2:**
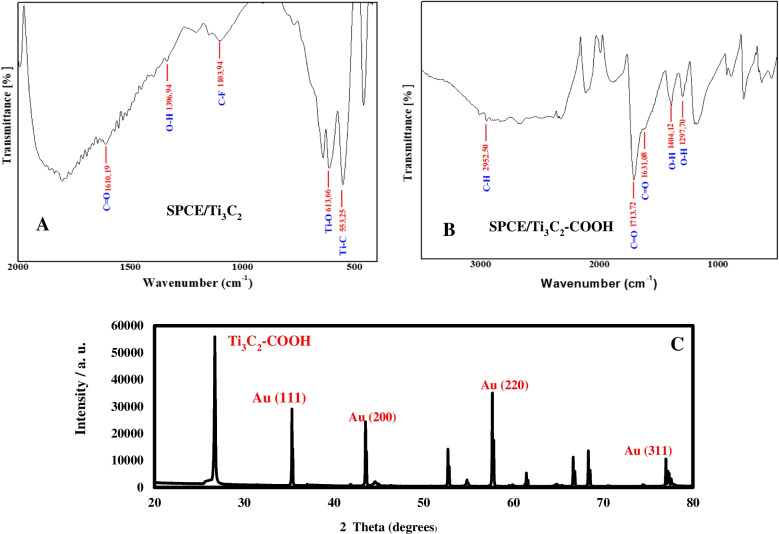
FTIR spectra of SPCE/Ti_3_C_2_ (**A**) and SPCE/Ti_3_C_2_-COOH (**B**). XRD spectra of SPCE/Ti_3_C_2_-COOH/AuNP (**C**)

FTIR spectra of SPCE/Ti_3_C_2_ and SPCE/Ti_3_C_2_-COOH are shown in Fig. 2A-B. The peak at 1610.19 cm^−1^ in the spectrum is the characteristic peak of C=O of Ti_3_C_2_ (Fig. 2A). The peaks at 1396.94 cm^−1^, 1103.94 cm^−1^, 613.66 cm^−1^, and 553.25 cm^−1^ result from the vibrations of the bonds in the O-H, C-F, Ti-O, and Ti-C groups, respectively [58, 59]. The peak at 2952.50 cm^−1^ in the spectrum is due to the C-H asymmetric vibration. The peaks at 1713.72 cm^−1^, 1631.08 cm^−1^, 1404.12 cm^−1^, and 1279.70 cm^−1^ are due to the vibration of the bonds in the C=O and -OH groups in the resonance structure of the carboxyl group (Fig. 2B) [58]. FTIR analysis results indicate that SPCE/Ti_3_C_2_ and SPCE/Ti_3_C_2_-COOH electrodes were successfully prepared.

#### XRD

XRD analysis results of SPCE/Ti_3_C_2_-COOH/AuNP are given in Fig. 2C. It shows the characteristic diffraction peak position (008) at 26.9° for Ti_3_C_2_-COOH (JCPDS no.: 032–1383). It has been reported that the characteristic diffraction peak position (008) for Ti_3_C_2_-COOH is observed at 28.80° [52]. Four characteristic diffraction peaks corresponding to the (111), (200), (220), and (311) planes for AuNP (JCPDS no.: 04–0784) were identified at 35.0°, 43.45°, 61.62°, and 77.58°, respectively [53–55]. XRD analysis indicated that SPCE/Ti_3_C_2_-COOH/AuNP electrodes were successfully prepared.

### Electrochemical characterizations of Ti_3_C_2_, Ti_3_C_2_-COOH, and AuNP modified electrodes

**Fig. 3:**
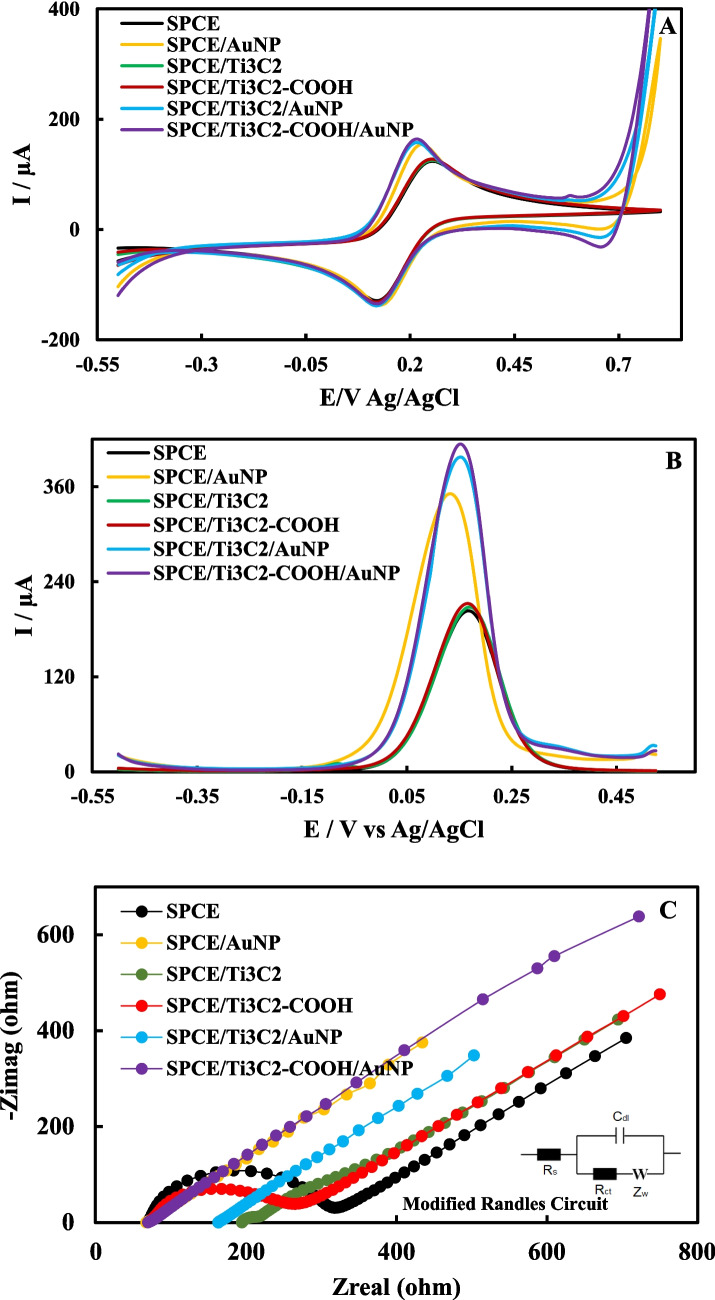
CVs (**A**), DPVs (**B**), and EISs (**C**) of SPCE, SPCE/AuNP, SPCE/Ti_3_C_2_, SPCE/Ti_3_C_2_-COOH, SPCE/Ti_3_C_2_/AuNP, and SPCE/Ti_3_C_2_-COOH/AuNP electrode

The electrochemical performance of the modified electrode directly affects the analytical performance of the HE4 immunosensor. To examine the effect of Ti_3_C_2_ and Ti_3_C_2_-COOH on the electrochemical performance of electrodes and immunosensors, the first step was to prepare SPCE/Ti_3_C_2_ and SPCE/Ti_3_C_2_-COOH. Subsequently, the modified electrodes were coated with AuNPs. The electrochemical characterization of SPCE, SPCE/AuNP, SPCE/Ti_3_C_2_, SPCE/Ti_3_C_2_-COOH, SPCE/Ti_3_C_2_/AuNP, and SPCE/Ti_3_C_2_-COOH/AuNP in 5 mM redox probe solution was performed using CV, DPV, and EIS. Voltammograms and electrochemical impedance spectra are shown in Fig. 3A–C. Table 1 shows the anodic peak currents (Ipa) and anodic peak current differences (ΔIpa) obtained from CV and DPV, and the resistance (Rct) and resistance difference (ΔRct = Rct_modified_− Rct_bare_) values obtained from EIS.

**Table 1:** Ipa, ΔIpa, and Aea values acquired from CV and DPV; Rct and ΔRct values obtained from EIS of SPCE, SPCE/AuNP, SPCE/Ti_3_C_2_, SPCE/Ti_3_C_2_-COOH, SPCE/Ti_3_C_2_/AuNP, and SPCE/Ti_3_C_2_-COOH/AuNP

**Formulation**	**CV**		**DPV**		**EIS**		**Aea (cm**^**2**^**)**
	**Ipa (µA)**	**ΔIpa (µA)**	**Ipa (µA)**	**ΔIpa (µA)**	**Rct (ohm)**	**ΔRct (ohm)**	
SPCE	127.94	-	202.47	-	233.30	**-**	0.1400
SPCE/AuNP	145.62	+ 17.68	332.39	+ 129.92	58.64	174.96	0.1593
SPCE/Ti_3_C_2_	128.92	+ 0.98	203.98	+ 1.53	181.20	52.40	0.1410
SPCE/Ti_3_C_2_-COOH	131.08	+ 3.14	210.35	+ 7.98	32.95	200.65	0.1468
SPCE/Ti_3_C_2_/AuNP	151.11	+ 23.17	377.64	+ 175.17	66.82	114.38	0.1653
SPCE/Ti_3_C_2_-COOH/AuNP	156.36	+ 28.42	394.95	+ 192.48	2.39	30.56	0.1710

The Ti_3_C_2_ and Ti_3_C_2_-COOH nanomaterials were tested to determine which of the two would provide a relative improvement in the electrode’s apparent electrochemical activity. For this purpose, the electroactive surface areas (Aea) of the modified electrodes were calculated using the Randles-Sevcík equation ($Ip=2.69\times {10}^{5}{n}^{3/2}AeaC\surd D\vartheta$), which is frequently used in electrochemical sensor and biosensor studies; the calculated Aea values are given in Table 1 [60]. According to the CV results, an increase of 0.98 µA (ΔI) in Ipa was observed when SPCE was modified with Ti_3_C_2_, and an increase of 3.14 µA (ΔI) in Ipa was noticed when SPCE was modified with Ti_3_C_2_-COOH. Ti_3_C_2_-COOH increased the conductivity more than Ti_3_C_2_. In DPVs, an increase of 1.53 µA (ΔIp) was observed in the SPCE/Ti_3_C_2_, and 7.98 µA (ΔIp) in the SPCE/Ti_3_C_2_-COOH. The Aea value of the SPCE is 0.1400 cm^2^. After SPCE/Ti_3_C_2_ and SPCE/Ti_3_C_2_-COOH modifications, the Aea increased to 0.1410 cm^2^ and 0.1468 cm^2^, respectively. Shankar et al. calculated the Aea as 0.0015 cm^2^ for the carbon electrode and 0.0129 cm^2^ for the MXene-doped carbon electrode. It was emphasized that the MXene-doped carbon electrode increased the Aea and electrochemical activity [61]. Similar results were obtained in our study. In EISs, the Rct decreased by 52.40 ohms (ΔRct) with the Ti_3_C_2_ modification on SPCE, while the Rct decreased by 200.65 ohms (ΔRct) with the Ti_3_C_2_-COOH modification. Electrochemical measurement results indicate that the Ti_3_C_2_-COOH modification exhibits higher conductivity compared to Ti_3_C_2_. After modification with AuNP, Ipa values increased for all electrodes in CVs. For SPCE/AuNP, SPCE/Ti_3_C_2_/AuNP, and SPCE/Ti_3_C_2_-COOH/AuNP, the ΔIs are 17.68 µA, 23.17 µA, and 28.42 µA, respectively. The highest current increase was observed with the SPCE/Ti_3_C_2_-COOH/AuNP electrode. In DPVs, the ΔIs of 129.92 µA, 175.17 µA, and 192.48 µA were obtained for SPCE/AuNP, SPCE/Ti_3_C_2_/AuNP, and SPCE/Ti_3_C_2_-COOH/AuNP, respectively. In the EIS results, the ΔRct value in the SPCE/Ti_3_C_2_-COOH/AuNP is higher than in the SPCE/AuNP and SPCE/Ti_3_C_2_/AuNP. The electrochemical characterization results indicate that the electrode exhibiting the greatest conductivity is SPCE/Ti_3_C_2_-COOH/AuNP. In addition, SEM images characterize the morphological properties of the electrode surface, whereas electrochemical impedance spectra characterize the electrochemical response associated with that morphology. Rougher, more porous, well-dispersed, and thinner films observed in the SEM images of the SPCE/Ti_3_C_2_-COOH/AuNP electrode (Fig. 1E) are associated with a lower Rct in the EISs (Fig. 3C) [62]. According to EIS and SEM results, SPCE/Ti_3_C_2_-COOH/AuNP electrodes were successfully prepared. The fitted Rct value of 2.39 ± 0.28 Ω obtained for the SPCE/Ti_3_C_2_-COOH/AuNP electrode is well above the instrumental resolution and significantly lower than that of the other electrodes, confirming the highly facilitated interfacial electron transfer kinetics resulting from the synergistic effect of MXene sheets and Au nanoparticles.

The amount of nanomaterials on the electrode surface affects the analytical performance of the modified electrode and biosensor. Therefore, optimization studies were conducted to determine the optimal amount of Ti_3_C_2_-COOH and AuNP. In the optimization studies, CV measurements at various scan rates (10–125 mV s^−1^) and EIS measurements of the modified electrodes were performed in the 5 mM redox probe solution.

First, SPCE/Ti_3_C_2_-COOH electrodes with different layer numbers (0 LBL, 1 LBL, 2 LBL, and 3 LBL) were prepared. The Aea of the modified electrodes Ti_3_C_2_-COOH at different layer numbers was calculated using CVs and Ip vs. v^1/2^ graphs (Fig. S1A–H). The Aea values were 0.1252 cm^2^ (bare SPCE, 0 LBL), 0.1350 cm^2^ (SPCE/Ti_3_C_2_-COOH, 1 LBL), 0.1247 cm^2^ (SPCE/Ti_3_C_2_-COOH, 2 LBL), and 0.1130 cm^2^ (SPCE/Ti_3_C_2_-COOH, 3 LBL). The maximum Aea value was achieved with the electrode fabricated using 1 LBL Ti_3_C_2_-COOH. In electrodes modified with 2 LBL and 3 LBL Ti_3_C_2_-COOH, there is a higher amount of Ti_3_C_2_-COOH compared to 1 LBL, resulting in a thicker layer. Therefore, it is thought that increasing the number of layers reduces the transfer of electroactive species from the electrode surface to the solution. Since the highest relative improvement in the apparent electrochemical activity was obtained in the SPCE/Ti_3_C_2_-COOH (1 LBL) electrode, the optimum amount of Ti_3_C_2_-COOH was selected as 1 LBL. AFM analyses were performed to support the results of determining the optimum amount of Ti_3_C_2_-COOH by the electrochemical method with surface morphology. AFM images for SPCE/Ti_3_C_2_-COOH (1 LBL), SPCE/Ti_3_C_2_-COOH (2 LBL), and SPCE/Ti_3_C_2_-COOH (3 LBL) electrodes are added to the supplementary files as Fig. S2. In the AFM image of SPCE/Ti_3_C_2_-COOH (1 LBL), the surface is relatively flat and well-coated, and the morphology appears homogeneous (Fig. S2A). In SPCE/Ti_3_C_2_-COOH (2 LBL) and SPCE/Ti_3_C_2_-COOH (3 LBL), the surface is rougher, and there are significant height differences (Fig. S2B, C). As the number of layers increases, agglomeration and topographic height differences are observed on the surface [63]. Therefore, increasing the number of layers is thought to reduce the transfer of electroactive substances from the electrode surface to the solution. We think that the AFM analysis results and the electrochemical characterization results are compatible with each other.

Secondly, an optimization study was conducted to determine the optimal amount of AuNPs. The amount of AuNP is affected by the HAuCl_4_ concentration and the number of cycles in the CV. In the HAuCl_4_ concentration optimization study, AuNP deposition was performed using HAuCl_4_ solutions prepared at three different concentrations (2 mM, 4 mM, and 6 mM). The Aea of the SPCE/Ti_3_C_2_-COOH/AuNP prepared at different HAuCl_4_ concentrations was calculated using CVs and Ip vs. v^1/2^ graphs (Fig. S3A–F). The Aea of the SPCE/Ti_3_C_2_-COOH/AuNP was calculated as 0.1487 cm^2^ (2 mM), 0.1553 cm^2^ (4 mM), and 0.1678 cm^2^ (6 mM), and the maximum relative improvement in the apparent electrochemical activity was achieved in the electrode prepared with 6 mM HAuCl_4_ solution. To support the CV results, EIS measurements of the SPCE/Ti_3_C_2_-COOH/AuNP prepared at different HAuCl_4_ concentrations were made, and the EISs are given in Fig. S3G. The Rct values of the modified electrodes were 53.99 ohms (2 mM), 29.14 ohms (4 mM), and 19.02 ohms (6 mM), respectively. The lowest Rct value was obtained in the electrode prepared with 6 mM HAuCl_4_ solution. According to Aea and Rct values, the optimum concentration was chosen as 6 mM since the highest conductivity was obtained at 6 mM HAuCl_4_. To determine the optimum number of cycles, AuNP deposition was performed for 5, 10, and 15 cycles. The Aea of the SPCE/Ti_3_C_2_-COOH/AuNP prepared at different numbers of cycles was calculated using CVs and Ip vs. v^1/2^ graphs (Fig. S4A–F). The Aea of the modified electrodes was calculated as 0.1460 cm^2^ (5n), 0.1678 cm^2^ (10n), and 0.1773 cm^2^ (15n), and the highest relative improvement in the apparent electrochemical activity was obtained in the electrode prepared with 15 cycles. To support the CV results, EIS measurements of the SPCE/Ti_3_C_2_-COOH/AuNP prepared at different numbers of cycles were made, and the EISs are given in Fig. S4G. The Rct values of the electrodes were found to be 67.88 ohms (5n), 19.02 ohms (10n), and 10.98 ohms (15n). The optimal number of cycles was established at 15 n, based on the lowest Rct and highest Aea values.

### Electrochemical characterizations of HE4 immunosensors

**Fig. 4:**
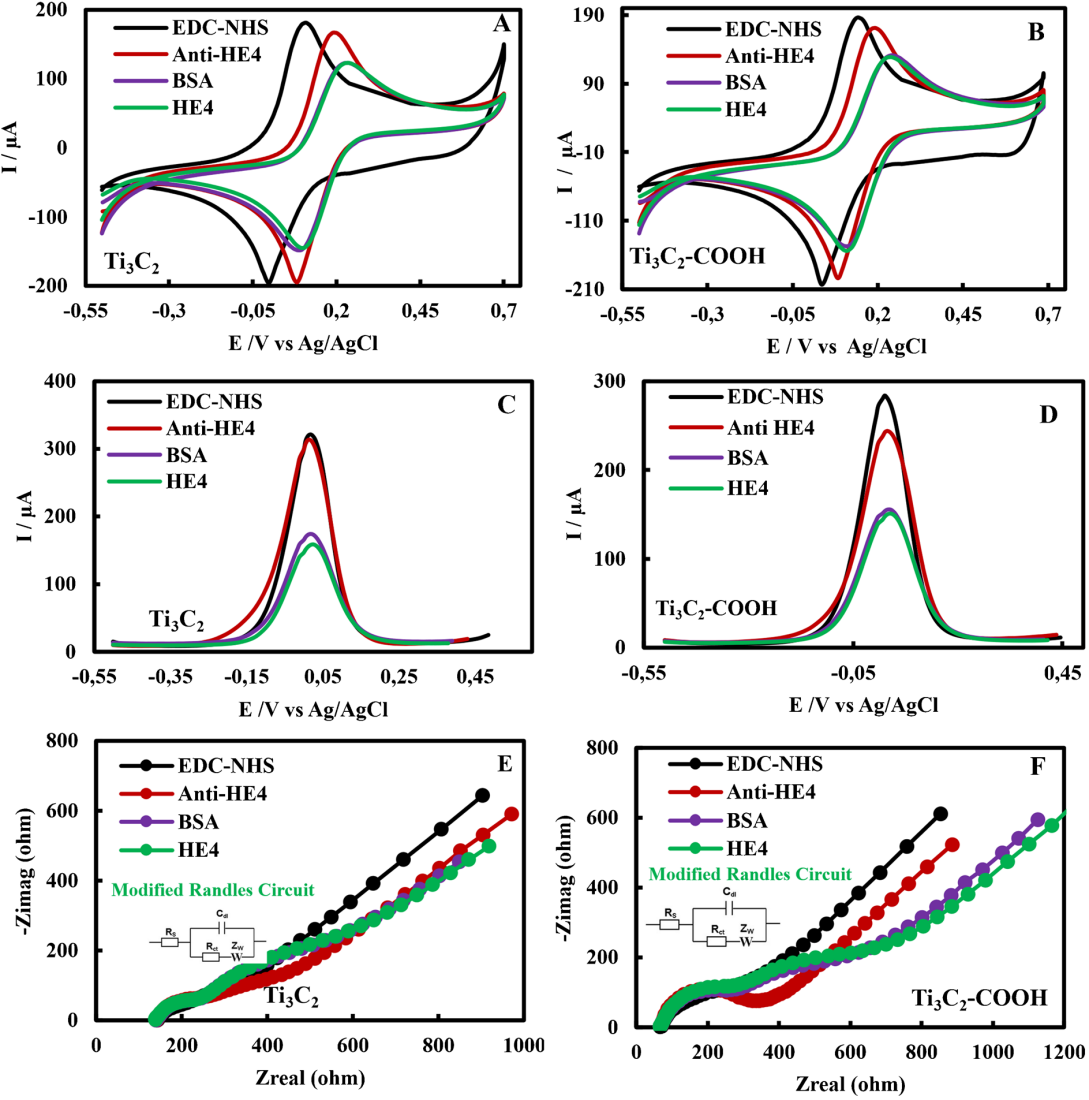
CVs (**A**, **B**), DPVs (**C**, **D**), and EISs (**E**, **F**) of the SPCE/Ti3C2/AuNP/Anti-HE4/BSA/HE4 and SPCE/Ti3C2-COOH/AuNP/Anti-HE4/BSA/HE4 immunosensors at each fabrication stage

In this section, HE4 immunosensors were fabricated using Ti_3_C_2_-AuNP and Ti_3_C_2_-COOH-AuNP modified SPCEs. The immunosensing mechanism and the effects of Ti_3_C_2_ and Ti_3_C_2_-COOH on immunosensor performance were investigated. Electrochemical characterization of the HE4 immunosensor preparation steps was performed using CV, DPV, and EIS in a 5 mM redox probe solution. CVs, DPVs, and EISs are shown in Fig. 4A–F, and Ipa, Epa, and Rct values are given in Table 2.

**Table 2:** Ipa_avg_ values acquired from CV and DPV, and Rct_avg_ values obtained from EIS of HE4 immunosensors fabricated with SPCE/Ti_3_C_2_/AuNP and SPCE/Ti_3_C_2_-COOH/AuNP

	**CV**	
	**SPCE/Ti**_**3**_**C**_**2**_**/AuNP (A)**	**SPCE/Ti**_**3**_**C**_**2**_**-COOH/AuNP (B)**
**Formulation**	**Ipa**_**avg**_** (µA)**	**Ipa**_**avg**_** (µA)**
EDC-NHS	156.78	162.16
Anti-HE4	155.34	157.75
BSA	116.44	121.54
HE4	114.47	120.31
	**DPV**	
	**SPCE/Ti**_**3**_**C**_**2**_**/AuNP (C)**	**SPCE/Ti**_**3**_**C**_**2**_**-COOH/AuNP (D)**
**Formulation**	**Ipa**_**avg**_** (µA)**	**Ipa**_**avg**_** (µA)**
EDC-NHS	308.22	276.22
Anti-HE4	300.33	231.59
BSA	160.47	147.71
HE4	148.15	144.02
	**EIS**	
	**SPCE/Ti**_**3**_**C**_**2**_**/AuNP (E)**	**SPCE/Ti**_**3**_**C**_**2**_**-COOH/AuNP (F)**
**Formulation**	**Rct**_**avg**_** (ohm)**	**Rct**_**avg**_** (ohm)**
EDC-NHS	137.70	99.88
Anti-HE4	265.50	286.70
BSA	427.00	830.80
HE4	495.10	871.10

To prepare HE4 immunosensors, 6-MHA was first applied to the surface of the AuNPs on the SPCE/Ti_3_C_2_-COOH/AuNP electrode to form homogeneous SAMs. 6-MHA attaches to the surface of AuNP via thiol groups, which helps keep the carboxyl (-COOH) functional groups properly oriented on the electrode. These carboxyl groups were then activated with EDC/NHS, making them suitable for covalent bond formation with biomolecules having an amine moiety (anti-HE4). A decrease in Ipa values during the anti-HE4 step, observed for both immunosensors in CV and DPV, and an increase in Rct values in EIS indicate that monoclonal anti-HE4 antibodies were successfully immobilized on the electrode surface. This change in electrochemical signals during the anti-HE4 steps occurs because anti-HE4 renders the electrode surface more insulating than in the EDC-NHS step, thereby reducing electron diffusion from the redox probe solution to the electrode surface [2, 49, 55]. After the binding of anti-HE4, the blank and active spots on the surface were incubated with BSA to prevent nonspecific binding. During this step, a thicker bioactive layer was formed on the electrode surface than during the anti-HE4 step, resulting in decreased diffusion, lower Ipa values, and higher Rct values. Specific binding of HE4 antigen to monoclonal anti-HE4 occurred after the BSA step. After incubation with HE4 antigens, the insulating layer on the electrode surface increased further compared with the BSA step [2, 49, 55]. These findings demonstrate the successful formation of an effective biorecognition layer on the immunosensor surface. In the HE4 immunosensor prepared with the SPCE/Ti_3_C_2_-COOH/AuNP electrode, the decline in the Ipa value and the rise in the Rct value of the antibody step are more pronounced than in the immunosensor prepared with the SPCE/Ti_3_C_2_/AuNP electrode. A critical stage in immunosensor design is the effective binding of antibodies to the surface. For this reason, HE4 immunosensors were fabricated using SPCE/Ti_3_C_2_-COOH/AuNP.

### Optimization studies of HE4 immunosensors

**Fig. 5:**
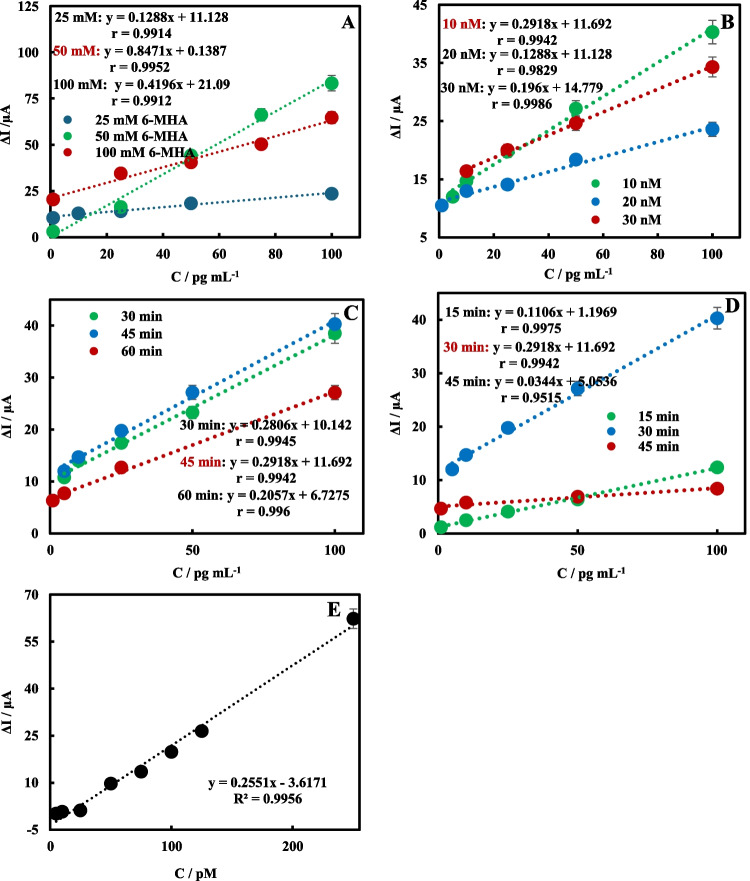
Optimization study of 6-MHA concentration (**A**), Anti-HE4 concentration (**B**), Anti-HE4 incubation time (**C**), HE4 incubation time (**D**), and ∆I vs. HE4 concentration plots (**E**)

Optimization studies are the most critical part of developing an immunosensor. Therefore, key steps in the preparation of the HE4 immunosensor were optimized. These studies included optimization of 6-MHA, of the concentration of anti-HE4, of the incubation time of anti-HE4, and of the HE4 antigen. The immunosensors prepared in the optimization studies were incubated with HE4. HE4 analysis was performed using DPV in a 1 mM redox probe solution. The graphs of HE4 concentrations versus peak current difference (ΔI = I_HE4_ − I_BSA_) values are given in Fig. 5A–-D. In a study to determine the optimum 6-MHA concentration for generating SAMs on the surface of AuNPs, immunosensors were prepared with three different 6-MHA concentrations (25 mM, 50 mM, and 100 nM). Immunosensors prepared by incubation with antigens were used to analyze HE4 antigens at different concentrations. ∆I-C plots were constructed for each immunosensor using the ∆I values (Fig. 5A). In Fig. 5A, the slope values ​​obtained from the HE4 analysis of immunosensors prepared with 25 mM and 100 mM 6-MHA are lower than those of immunosensors prepared with 50 mM 6-MHA. The highest slope (0.8471) and r value (0.9952) were obtained from the analysis of immunosensors prepared with 50 mM 6-MHA. Therefore, the optimum 6-MHA concentration was determined to be 50 mM. For the anti-HE4 concentration-optimization study, concentrations of 10 nM, 20 nM, and 30 nM anti-HE4 were tested (Fig. 5B). In Fig. 5B, the slope values ​​obtained from the HE4 analysis of immunosensors prepared with 20 nM and 30 nM Anti-HE4 were similar (0.1288 and 0.1960, respectively). The highest slope value (0.2918) was observed at 10 nM, which was determined to be the optimum concentration.

To optimize the anti-HE4 incubation time, we tested durations of 30, 45, and 60 min. In Fig. 5C, the slope of the ΔI-C graph obtained from the HE4 analysis of HE4 immunosensors prepared with a 60-min Anti-HE4 incubation (60 min: 0.2057) is lower than that of immunosensors prepared with 30- and 45-min incubations. The slope values of immunosensors prepared by 30- and 45-min incubations with Anti-HE4 are 0.2806 and 0.2918, respectively, which are similar. Although the highest r value was obtained from HE4 analysis of immunosensors prepared with a 60-min incubation, it was quite close to the r values obtained from HE4 analysis of immunosensors prepared with 30- and 45-min incubations. When the slope and r values are evaluated together, the optimum Anti-HE4 incubation time is 45 min. To optimize the incubation time for HE4, we tested durations of 15, 30, and 45 min. In Fig. 5D, despite the increase in the r-value at 15 min, low signals were still recorded. Furthermore, the slope values indicate that this was insufficient for antibody-antigen interaction. After a 30-min HE4 incubation, the slope was relatively high, and the r value increased. Consequently, a 30-min incubation time was selected as the optimum.

### Analytical performance of the HE4 immunosensors

To evaluate the analytical performance of the fabricated HE4 immunosensor, analysis of HE4 was carried out using SPCE/Ti_3_C_2_-COOH/AuNP/Anti-HE4/BSA immunosensors in a linear range of 5–250 pM in a 1 mM redox probe solution with a portable electrochemical reader. Figure 5E shows that the ΔI values increase as HE4 concentration rises. The sensitivities and detection limits (based on 3 σ/m) of the HE4 immunosensors are 0.2551 µA/pM and 0.34 pM, respectively. The **repeatability** of the HE4 immunosensors was tested with a 50 pM HE4 solution and was found to be consistent across seven tests (RSD%: 3.38%). Six immunosensors were manufactured and used for HE4 measurement (50 pM) on different days to assess **reproducibility** (Fig. S5A). The RSD% of response changes between immunosensors is 1.22%, indicating that the immunosensors consistently produce similar results. To test the integrity of the sensing surface of the prepared immunosensors, the antibody-antigen complex was dissociated and then reconstituted. For this process, HE4 immunosensors were first treated with 10 mM HCl for 2 min at room temperature and then incubated with HE4 (50 pM) for 30 min. This process was continued until an increase in signal, rather than a decrease, was observed in the DPV analyses after 3 cycles. Following the first and second immunocomplex separation procedures, 97.2% (n = 3, 33.75 (µA) ± 0.02) and 88.4% (n = 3, 30.71 (µA) ± 0.03) signal was observed, respectively (Figure S5B). In the fourth procedure, a 90.7% (n = 3, 31.51 (µA) ± 0.03) increase in immunosensor response was observed, and the immunosensing surface stability study was stopped. The signal increase observed after the fourth procedure suggests that partial denaturation of antibody-antigen complexes or rearrangement of the bioactive layer may temporarily increase electron transfer. These findings demonstrate that, despite their disposable nature, immunosensors perform well under stress testing for surface robustness.

To test the assess **stability**, HE4 immunosensors were prepared, and HE4 (50 pM) analyses were performed for and analyses of HE4 (50 pM) were performed over 60 days. Average relative ∆I% values were calculated by comparing them to the initial value and were plotted against the number of weeks (Fig. S5C). The response of the HE4 immunosensors decreased by 11.21% after 3 days, 14.62% after 5 days, 21.86% after 20 days, and 34.98% after 30 days compared to the initial value. Although the HE4 immunosensor signal decreased by 65.93% on day 60, it continued to respond to the HE4 antigen. To examine the **shelf life** of HE4 immunosensors, 10 immunosensors were fabricated to the BSA stage and stored at +4°C until week 32. Analyses of HE4 (50 pM) were performed using HE4 immunosensors at weeks 1, 2, 4, 6, 8, 12, 16, 20, and 24. From the analysis results, average relative ∆I% values were calculated by comparing them with the initial value and plotted against the number of weeks (Fig. S5D). The response of the HE4 immunosensors decreased by 9.49% after 1 week of storage, by 32.73% after 12 weeks, and by 61.62% after 20 weeks compared with the initial value. HE4 analysis was not performed for the 32-week storage period because a 78.01% signal decrease was observed after 24 weeks. One of the most important factors affecting the stability and shelf life of immunosensors is the material used to modify the electrode surface. The Ti_3_C_2_-COOH used in this study is a graphene-based material with a structure resistant to degradation. AuNPs, on the other hand, are known for their biocompatibility. The combined use of Ti_3_C_2_-COOH and AuNPs in this study is thought to increase the stability and shelf life of the HE4 immunosensor.

The selectivity of HE4 immunosensors was investigated in the presence of FDA-approved biomarkers of ovarian cancer, CA125 and alpha-fetoprotein (AFP), as well as potential biomarkers [2, 49, 55], anterior gradient-2 protein (AGR2), folate receptor alpha (FOLR1), glycodelin (GLY), and soluble mesothelin-associated protein (SMRP) antigens. For this purpose, samples were prepared in both pH 7.4 PBS and commercial blood serum (diluted 1:100 with PBS), each containing individual antigens, a mixture of all antigens, and a mixture of all antigens excluding the target antigen, each at 50 pM. HE4 immunosensors prepared up to the BSA step were incubated with the aforementioned samples, and DPV measurements were taken in redox probe solution. The graphs of the analysis results are shown in Figure S5E and F, and the corresponding data are provided in Table S2. In PBS (pH 7.4), the immunosensor response to individually prepared antigens was less than 3%, whereas the response to the mixture containing all antigens was 98.93% (Figure S5E, Table S2). In commercial blood serum, the immunosensor response for individually prepared antigens was approximately 5%, while the response for the mixture containing all antigens was 98.07% (Figure S5F, Table S2). These findings demonstrate that the developed HE4 immunosensors exhibit acceptable selectivity for HE4 in the presence of other ovarian cancer markers.

The analytical performance of different label-free electrochemical HE4 immunosensors is given in Table 3. Some of the studies in Table 3 [36, 37, 39] tested the HE4 immunosensors they developed using clinical samples from ovarian cancer patients. HE4 analyses were also performed on samples from patients using ELISA. However, these HE4 immunosensors used in the analysis of real patient samples are based on SPR, LSPR, and PEC. Other HE4 immunosensors listed in Table 3 are electrochemical. HE4 analysis was not performed on samples from real patients using electrochemically based HE4 immunosensors. This suggests that electrochemical immunosensors may have limitations for antigen analysis in real patient samples. In our study, HE4 analysis was also not performed on real patient samples with the disposable electrochemical HE4 immunosensors we developed because we did not have the opportunity to work with real patients. However, we believe that the method we proposed and the HE4 immunosensors we developed may be suitable for direct HE4 analysis of real patient samples and for POCT. Moreover, compared to other studies, none of the label-free electrochemical HE4 immunosensors directly measured HE4 levels at pM concentrations. As summarized in Table 3, the proposed SPCE/Ti_3_C_2_-COOH/AuNP-based immunosensor exhibits a competitive limit of detection (0.34 pM) while maintaining a clinically relevant linear range (5–250 pM). Compared to previously reported HE4 immunosensors, many of which either show extremely low detection limits with narrow working ranges or require complex and costly measurement systems, the present sensor offers a balanced combination of sensitivity, a practical linear range, and a simple, label-free electrochemical platform. This balance highlights its potential for routine and point-of-care HE4 determination. The HE4 immunosensors we propose in our study are cost-effective and provide practical measurements of HE4 levels without requiring expert assistance. The HE4 immunosensors we developed appear promising for the early diagnosis of ovarian cancer.

**Table 3:** Analytical performances of different label-free electrochemical HE4 immunosensors

Label-free HE4 immunosensors	LOD	Detection range	Method	Ref
Glass surface/AgNP/11-MUA/EDC-NHS/AntiHE4/HE4	4 pM	10–10,000 pM	LSPR	[36]
GCE/ILWS_2_/HGNs/AntiHE4/BSA/HE4 Ag	2.7 pM	0.009–909 pM	PEC	[37]
GCE/TiO_2_-NGO/Au@Pd HS/AntiHE4/BSA/HE4 Ag	13.33 fM	10 fM–60 nM	CA	[33]
GCE/TOAB-GE/C_60_/AntiHE4/BSA/HE4	18.2 pM	90.9–9090 pM	DPV	[38]
Gold chip/systemamine/EDC-NHS/AntiHE4/BSA/HE4	2 pM	2–120 pM	SPR	[39]
GCE/PtNi NCAs/AntiHE4/BSA/HE4	0.010 pM	0.91–9090 pM	DPV	[64]
FTO/CdS NS/AuNP/AntiHE4/BSA/HE4	0.108 pM	0.91–18 180 pM	EC, PEC	[40]
SPCE/RGO/PTH/AuNP/AntiHE4/BSA/HE4	0.182 pM 12.7 pM 106 pM 757 pM 0.0909 pM 5.27 pM 120 pM 1.03 nM 0.182 pM 14.9 pM 202 pM 897 pM	0.0909–9.09 pM 0.91–909 pM 909–4545 pM 4545–45,450 pM 0.0909–9.09 pM 4.55–227 pM 909–4545 pM 4545–45,450 pM 0.0909–9.09 pM 9.09–909 pM 909–4545 pM 4545–45,450 pM	EIS DPV SWV	[2]
SPCE/RGO/PTH/AuNP/AntiHE4/BSA/HE4	0.00527 pM 120 pM 0.0149 pM 202 pM	0.0909–9.09 pM 90.9–4545 pM 0.0909–9.09 pM 90.9–4545 pM		[49]
ITO-OH/3-APTES/GA/AntiHE4/BSA/HE4	0.00855 pM	0.0909–273 pM	CV, EIS	[41]
GCE/PT/CS/MPC/AntiHE4/BSA/HE4	0.256 pM	0.0909–909 pM	DPV	[65]
SPE/WS_2_@PANI/AntiHE4/BSA/HE4	0.00136 pM	0.0091–45,450 pM	CV	[66]
**SPCE/Ti**_**3**_**C**_**2**_**-COOH/AuNP/Anti-HE4/BSA/HE4**	**0.34 pM**	**5–250 pM**	**DPV**	**This work**

*3-APTES*, 3-aminopropyltriethoxysilane; *11-MUA*, 11-mercapto andecanoic acid; *AgNP*, silver nanoparticle; *Au@Pd HS*, Au@Pd holothurian shaped nanoparticles; *AuNP*, gold nanoparticles; *C60*, Fullorene 60; *CA*, chronoamperometry; *CS*, chitosan; *CD-Ab*, carbon dot-Antibody; *CdS* *NS*, cadmium sulfur nanolayer; *DPV*, differential pulse voltammetry; *ECL*, electrochemiluminescence; *EDC-NHS*, N-ethyl-N′(3-dimethylaminopropyl) carbonimide-N-hydroxysuccinimide; *FTO*, fluorine-doped tin oxide; *GA*, glutaraldehyde; *GCE*, glassy carbon electrode; *HGNs*, hollow gold nanoparticles; *IL-WS2*, tungsten disulfide nanosheet-functionalized ionic liquid; *ITO*, indium tin oxide; *LSPR*, localized surface plasma resonance; *MPC*, MoS_2_@PDA@CuCoFe_2_O_4_; *PANI*, polyaniline; *PEC*, photoelectrochemical; *PT*, thiophene; *PTH*, polythionine; *PtNi NCAs*, platinum nickel nanocubes; *RGO*, reduced graphene oxide; *SPCE*, screen-printed carbon electrode; *SPR*, surface plasma resonance; *SWV*, square wave voltammetry; *TiO*_*2*_*-NGO*, nitrogen-doped reduced graphene oxide functionalized titanium oxide; *TOAB-GE*, tetraoctylammonium bromide-graphene; *WS*_*2*_, tungsten disulfide nanosheets

### HE4 analysis at pM levels in commercial human blood serum samples with a portable electrochemical reader and ELISA kits

HE4 analysis was performed at pM concentration in commercial human blood serum samples using the developed immunosensors, together with a portable electrochemical reader (DropStat). Firstly, we transferred the calibration graphs obtained from HE4 analysis using the DPV method with a portable electrochemical reader to Metrohm Dropsens Firm. The firm produced USB cards for calibration, installed the software, and sent them to us for use in real-sample analyses. Commercial human blood serum samples (Sigma H6914) were purchased, diluted 1:100 with pH 7.4 PBS, and then spiked with a known amount (50 pM) of HE4 to prepare spiked human serum samples [45, 55]. Serum samples containing HE4 antigen were incubated with the developed immunosensors, and HE4 analyses were performed in the 1 mM redox probe solution. HE4 levels in commercial human blood serum were read directly from the screen in pM concentrations using prepared immunosensors (preparation time: 105 min) and a portable electrochemical reader (device reading time 20–30 s) (Figure S6). In the analysis of HE4 in commercial human blood serum, the % recovery and % error were calculated to be 100.71% and 0.71%, respectively. HE4 levels in blood serum used for the early detection of ovarian cancer can be easily determined directly at pM concentrations using portable HE4 immunosensors coupled to an electrochemical reader. Therefore, the proposed HE4 immunosensors are candidates for use in POCT.

The accuracy of the HE4 analysis performed using HE4 immunosensors was compared with that performed using the reference method, the ELISA kit. A serum sample was prepared by adding 25 pM of HE4 antigen serum diluted 1:100 using the standard addition method. The HE4 antigen concentration was calculated from the absorbance data measured by UV/VIS and the ELISA kit’s calibration curve. The results of the HE4 analyses performed with the ELISA kit and the prepared HE4 immunosensor were statistically compared using the t-test (Table 4). According to Table 4, the p-values for the calculated t-test are less than 0.05; the calculated t-value for the HE4 immunosensor (0.27) is lower than the critical t-value (4.30). This result indicates that HE4 analyses obtained with the developed HE4 immunosensors are statistically consistent with those obtained by ELISA, with no significant difference between them. The accuracy of the developed HE4 immunosensors has been verified by ELISA.

**Table 4:** Comparison of HE4 analysis results performed with ELISA and HE4 immunosensors using t-tests

Added HE4 cons	Dropstat (pM)	ELISA (pM)	t_critical_	t	p
25 pM	24.51/24.54/24.56	24.51/24.41/24.76	4.3	0.27	0.811

## Conclusion

Label-free, practical, portable, POCT-candidate electrochemical HE4 immunosensors have been developed for the determination of HE4, an ovarian cancer biomarker. The performance of SPCE/Ti_3_C_2_ and SPCE/Ti_3_C_2_-COOH in transducers and electrochemical HE4 immunosensors was investigated, and Ti_3_C_2_-COOH and AuNP were found to exhibit synergistic effects. The combined use of Ti_3_C_2_-COOH and AuNP improved the stability and shelf life of the fabricated HE4 immunosensor and showed good reproducibility and repeatability.

The portable electrochemical HE4 immunosensor exhibits high specificity for HE4 in complex environments containing multiple ovarian cancer markers. Portable HE4 immunosensors used to analyze HE4 in commercial human blood serum samples have achieved recovery rates of over 99%. The accuracy of the immunosensor platform we produce has been verified using the standard ELISA method. The direct detection of HE4 antigen at pM concentrations in blood serum samples using portable immunosensors, which exhibit a wide linear range, a low detection limit, and a total analysis time of 105 min, demonstrates the potential to develop a method to detect other targeted cancer biomarkers.

Device miniaturization and integration into a handheld electrochemical reader could enable in-field measurements while minimizing required sample volume. This reliable system, exhibiting full agreement with reference methods and offering high reproducibility, forms a strong foundation for next-generation biosensor technologies that enable rapid, sensitive, and selective diagnosis of various cancer biomarkers.

## Supplementary Information

Below is the link to the electronic supplementary material.

Supplementary file1 (DOCX 4.43 MB)

## Data Availability

The authors declare that all data supporting the findings of this study are available in the article and supplementary materials.
