# Supplementary material for: Portable electrochemical immunosensor for point-of-care testing of ovarian cancer biomarker HE4 in commercial human blood serum samples
Source: Anal Bioanal Chem. 2026 Mar 29;418(11):3457–74. doi: 10.1007/s00216-026-06457-7 (PMC13197341; doi:10.1007/s00216-026-06457-7)
Supplement: Supplementary file 1 — Supplementary file1 (DOCX 4.43 MB) [file 216_2026_6457_MOESM1_ESM.docx]

**Supplementary Material**

**Portable electrochemical immunosensor for point-of-care testing of ovarian cancer biomarker HE4 in commercial human blood serum samples**

Merve YILMAZ ÇILÇAR^1^, Melike BİLGİ KAMAÇ^1*^

^1^Çankırı Karatekin University, Faculty of Science, Chemistry Department, Çankırı, Turkey

*Corresponding author. Çankırı Karatekin University, Faculty of Science, Chemistry Department, Çankırı, 18100, Turkey

*E-mail addresses:* melikesahin@karatekin.edu.tr

**Figure Captions**

**Fig S1.** CVs of the different Ti_3_C_2_-COOH layers (0 LBL, 1 LBL, 2 LBL and 3 LBL) SPCE/Ti_3_C_2_-COOH in 5 mM redox probe solution at different scan rates (10-25-50-75-100-125 mV s^-1^) **(A, B, C, D)**, Ipa-*v*^1/2^ graphs **(E, F, G, H)**.

**Fig S2.** AFM images for SPCE/Ti_3_C_2_-COOH (1 LBL), SPCE/Ti_3_C_2_-COOH (2 LBL), and SPCE/Ti_3_C_2_-COOH (3 LBL) electrodes

**Fig S3.** CVs of the different HAuCl_4_ concentrations (2 mM, 4 mM, and 6 mM) SPCE/Ti_3_C_2_-COOH/AuNP in 5 mM redox probe solution at different scan rates (10-25-50-75-100-125 mV s^-1^) **(A, B, C)**, Ipa-*v*^1/2^ graphs **(D, E, F)**

**Fig. S4.** CVs of the different cycle number AuNP-modified SPCEs in redox probe solution at different scan rates (10-25-50-75-100-125 mV s^-1^) **(A, B, C)**, Ipa-*v*^1/2^ graphs **(D, E, F)**

**Fig. S5.** Reproducibility study **(A),** Reusability study **(B),** Stability study **(C),** Shelf life study **(D),** Selectivity study (PBS) **(E),** and Selectivity study (Blood serum) **(F)**

**Fig. S6.** Photograph displayed during measurement of 100 pM HE4 using HE4 immunosensors and the hand-held electrochemical reader


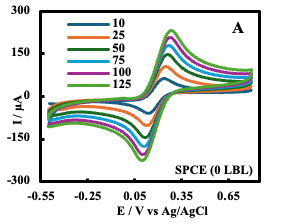

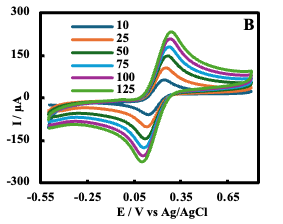

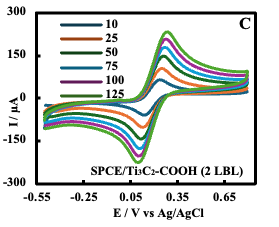

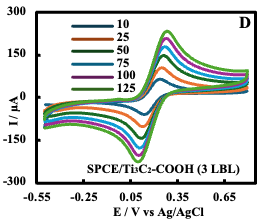

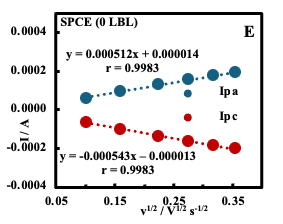

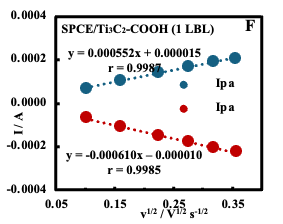

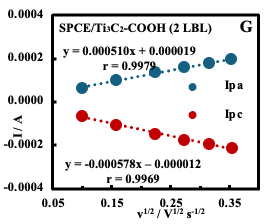

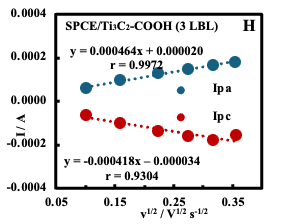


**SPCE/Ti_3_C_2_-COOH (1 LBL)**

**Fig S1.**

**
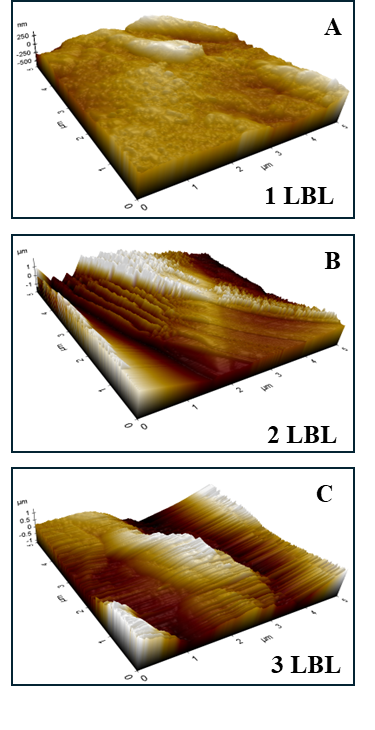
**

**Fig S2.**


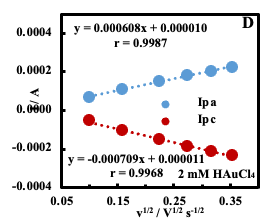

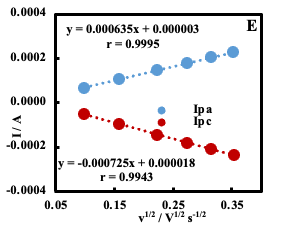


**4 mM HAuCl_4_**


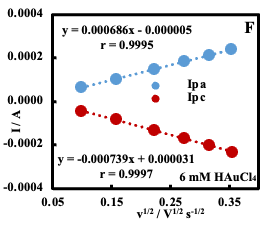

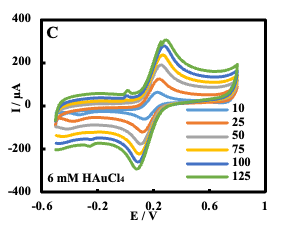

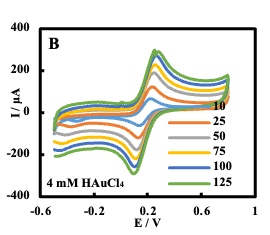

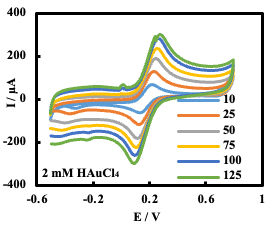


**A**


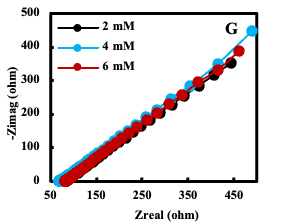

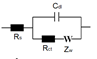


**A**

**Fig S3.**


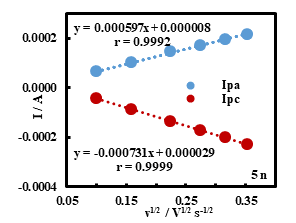

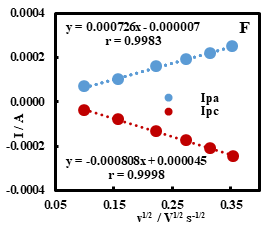


**15 n**


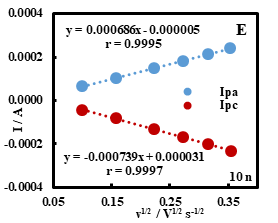

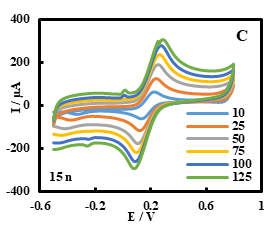

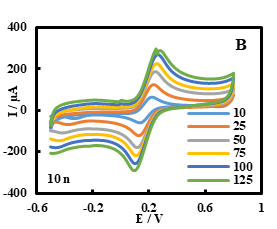

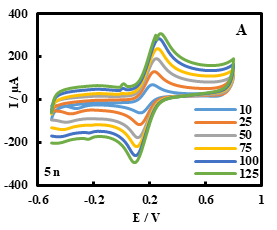


**D**


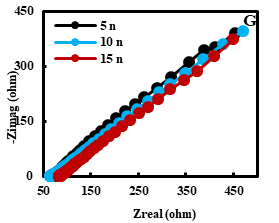

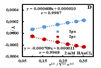


**Fig S4.**


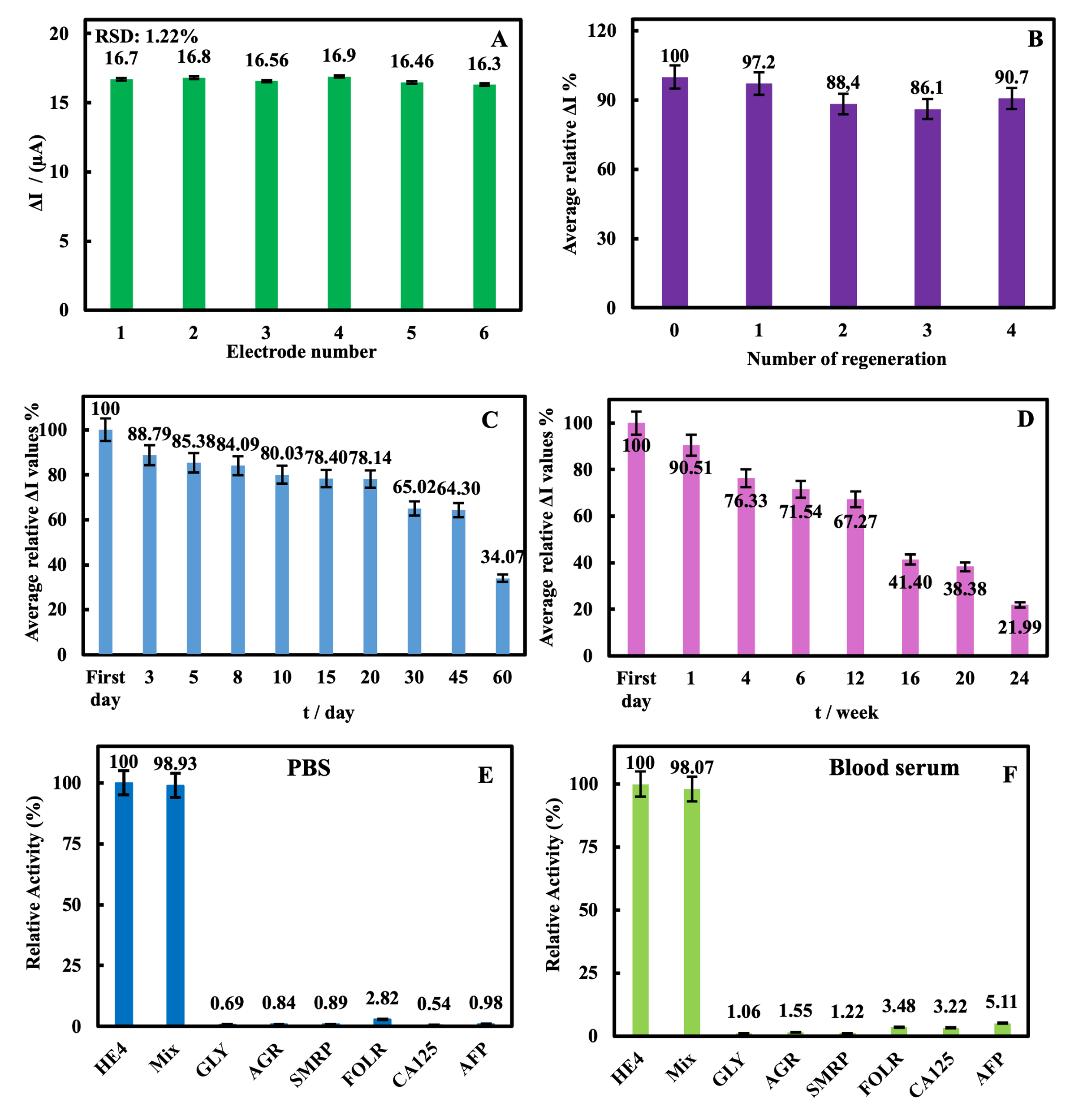


**Fig S5.**


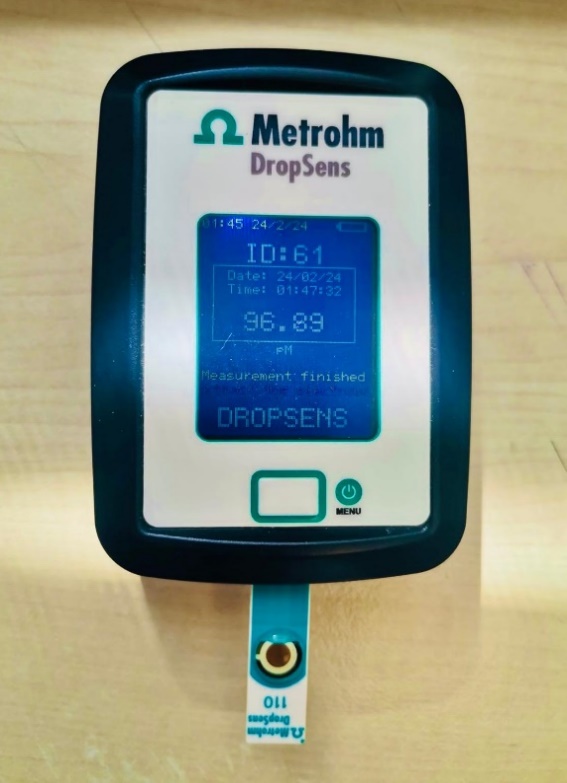


**Fig S6.**

**Table S1.** Rs, Rct, Cdl/CPE, W values obtained from EIS of SPCE, SPCE/AuNP, SPCE/Ti_3_C_2_, SPCE/Ti_3_C_2_-COOH, SPCE/Ti_3_C_2_/AuNP, SPCE/Ti_3_C_2_-COOH/AuNP

| **Electrode** | **Rs (Ω)** | **Rct (Ω)** | **CPE-Q (Ω⁻¹·sⁿ)** | **W (Ω·s⁻¹ᐟ²)** |
| --- | --- | --- | --- | --- |
| SPCE | 86.3 ± 2.1 | 233.3 ± 12.5 | (3.1 ± 0.3) ×10⁻⁵ | 215 ± 18 |
| SPCE/AuNP | 78.9 ± 1.9 | 58.6 ± 4.3 | (4.8 ± 0.4) ×10⁻⁵ | 142 ± 12 |
| SPCE/Ti₃C₂ | 81.5 ± 2.0 | 181.2 ± 10.1 | (3.6 ± 0.3) ×10⁻⁵ | 198 ± 15 |
| SPCE/Ti₃C₂-COOH | 74.2 ± 1.7 | 32.9 ± 3.1 | (5.9 ± 0.5) ×10⁻⁵ | 121 ± 10 |
| SPCE/Ti₃C₂/AuNP | 70.6 ± 1.6 | 66.8 ± 5.0 | (5.2 ± 0.4) ×10⁻⁵ | 134 ± 11 |
| SPCE/Ti₃C₂-COOH/AuNP | 62.4 ± 1.5 | 2.39 ± 0.28 | (7.4 ± 0.6) ×10⁻⁵ | 82 ± 7 |

**Table S2.** Selectivity test results

| **PBS (50 mM pH 7.4)** | | | | | |
| --- | --- | --- | --- | --- | --- |
| **Analyte** | **Concentration (pM)** | **n** | **mean±SD** | **% Relative response** | **Acceptance criterion** |
| **HE4** | 50 | 3 | 89.27**±**0.29 | 100 | √ |
| **mix** | 50 (each) | 3 | 88.50**±**0.44 | 98.93 | √ |
| **GLY** | 50 | 3 | 0.63**±**0.05 | 0.69 | √ |
| **AGR** | 50 | 3 | 0.81**±**0.06 | 0.84 | √ |
| **SMRP** | 50 | 3 | 0.88**±**0.06 | 0.89 | √ |
| **FOLR** | 50 | 3 | 2.54**±**0.09 | 2.82 | √ |
| **CA125** | 50 | 3 | 0.46**±**0.02 | 0.54 | √ |
| **AFP** | 50 | 3 | 0.83**±**0.04 | 0.98 | √ |
| **SERUM (1:100 commercial human serum:50 mM pH 7.4)** | | | | | |
| **Analyte** | **Concentration (pM)** | **n** | **mean±SD** | **% Relative response** | **Acceptance criterion** |
| **HE4** | 50 | 3 | 98.23**±**0.25 | 100 | √ |
| **mix** | 50 (each) | 3 | 96.73**±**0.42 | 98.07 | √ |
| **GLY** | 50 | 3 | 1.05**±**0.03 | 1.06 | √ |
| **AGR** | 50 | 3 | 1.57**±**0.04 | 1.55 | √ |
| **SMRP** | 50 | 3 | 1.20**±**0.09 | 1.22 | √ |
| **FOLR** | 50 | 3 | 3.37**±**0.06 | 3.48 | √ |
| **CA125** | 50 | 3 | 3.43**±**0.17 | 3.22 | √ |
| **AFP** | 50 | 3 | 5.15**±**0.08 | 5.11 | √ |
